# Plasminogen Activator Inhibitor-1 Protects Mice Against Cardiac Fibrosis by Inhibiting Urokinase-type Plasminogen Activator-mediated Plasminogen Activation

**DOI:** 10.1038/s41598-017-00418-y

**Published:** 2017-03-23

**Authors:** Kamlesh K. Gupta, Deborah L. Donahue, Mayra J. Sandoval-Cooper, Francis J. Castellino, Victoria A. Ploplis

**Affiliations:** 1 W. M. Keck Center for Transgene Research, University of Notre Dame, Notre Dame, IN USA; 2 Department of Chemistry and Biochemistry, University of Notre Dame, Notre Dame, IN USA

## Abstract

Plasminogen activator inhibitor-1 (PAI-1) is known to protect mice against cardiac fibrosis. It has been speculated that PAI-1 may regulate cardiac fibrosis by inactivating urokinase-type plasminogen activator (uPA) and ultimately plasmin (Pm) generation. However, the *in vivo* role of PAI-1 in inactivating uPA and limiting the generation of Pm during cardiac fibrosis remains to be established. The objective of this study was to determine if the cardioprotective effect of PAI-1 is mediated through its ability to directly regulate urokinase -mediated activation of plasminogen (Pg). An Angiotensin II (AngII)-aldosterone (Ald) infusion mouse model of hypertension was utilised in this study. Four weeks after AngII-Ald infusion, PAI-1-deficient (PAI-1^−/−^) mice developed severe cardiac fibrosis. However, a marked reduction in cardiac fibrosis was observed in PAI-1^−/−^/uPA^−/−^ double knockout mice that was associated with reduced inflammation, lower expression levels of TGF-β and proteases associated with tissue remodeling, and diminished Smad2 signaling. Moreover, total ablation of cardiac fibrosis was observed in PAI-1^−/−^ mice that express inactive plasmin (Pm) but normal levels of zymogen Pg (PAI-1^−/−^/Pg^S743A/S743A^). Our findings indicate that PAI-1 protects mice from hypertension-induced cardiac fibrosis by inhibiting the generation of active Pm.

## Introduction

Cardiac fibrosis, a stiffening of the cardiac tissue with resultant diminishment of heart function, leads to a variety of heart diseases^1^. Excessive synthesis and deposition of extracellular matrix proteins, such as collagen, in heart tissues are the principal causes of cardiac fibrosis^2^, yet mechanisms that regulate this outcome remain poorly understood. It is well established that growth factors and hormones, such as transforming growth factor-β (TGF-β) and angiotensin II (AngII), are primarily involved in enhancing synthesis of collagens and other matrix proteins and, as a result, have been implicated in the development of cardiac fibrosis^2, 3^. Enhanced expression and increased activation of profibrotic TGF-β in myocardium have been identified in experimental and human cardiovascular diseases and are associated with cardiac fibrosis and hypertrophy^3, 4^. Previous studies have shown that immune neutralisation of TGF-β1 or deletion of its signaling partner, Smad3, reduced the extent of cardiac fibrosis in mice^4, 5^, whereas transgenic mice overexpressing TGF-β1 develop severe cardiac fibrosis^6, 7^. TGF-β is secreted as a latent complex which is unable to associate with its receptors (TβRI/II). Activation of TGF-β requires proteolytic cleavage and separation of its N-terminal pro-domain, LAP (TGF-β latency-associated peptide) by plasma membrane-bound furin, or other extracellular proteases such as plasmin (Pm) and matrix metalloproteinases (MMP-2 and MMP-9)^8, 9^.

Mounting evidence implicates a critical involvement of coagulation and fibrinolysis proteins in cardiac fibrosis. For example, mice overexpressing urokinase-type plasminogen activator (uPA) in macrophages develop spontaneous cardiac fibrosis^10^, whereas the loss of uPA protects mice against cardiac fibrosis in response to myocardial infarction^11^. Further, mice deficient for PAI-1 were shown to develop cardiac fibrosis under both nonhypertensive (spontaneous) and hypertensive (AngII-induced) conditions^12–15^. It is believed that the elevated cardiac fibrosis in these animal models is primarily driven by increased uPA-mediated activation of plasminogen (Pg), leading to an overall increase in TGF-β activation and signaling. It has been speculated that PAI-1 may regulate cardiac fibrosis by inactivating uPA, thereby limiting Pm generation^16^. However, the *in vivo* role of inhibiting uPA function by PAI-1, and its subsequent effects on Pm generation during cardiac fibrosis is not well established.

In the current study, we sought to test the hypothesis that the cardiac fibrosis phenotypes observed in PAI-1^−/−^ mice will be alleviated by the complete absence of either uPA or active Pm. For this, we employed an AngII-aldosterone-induced model of cardiac fibrosis in mice with targeted deletion of components of the fibrinolytic system. The results of this study are summarized herein.

## Results

### The severity of AngII-Ald-induced cardiac fibrosis is reduced in PAI-1^−/−^/uPA^−/−^ mice

**Figure 1:**
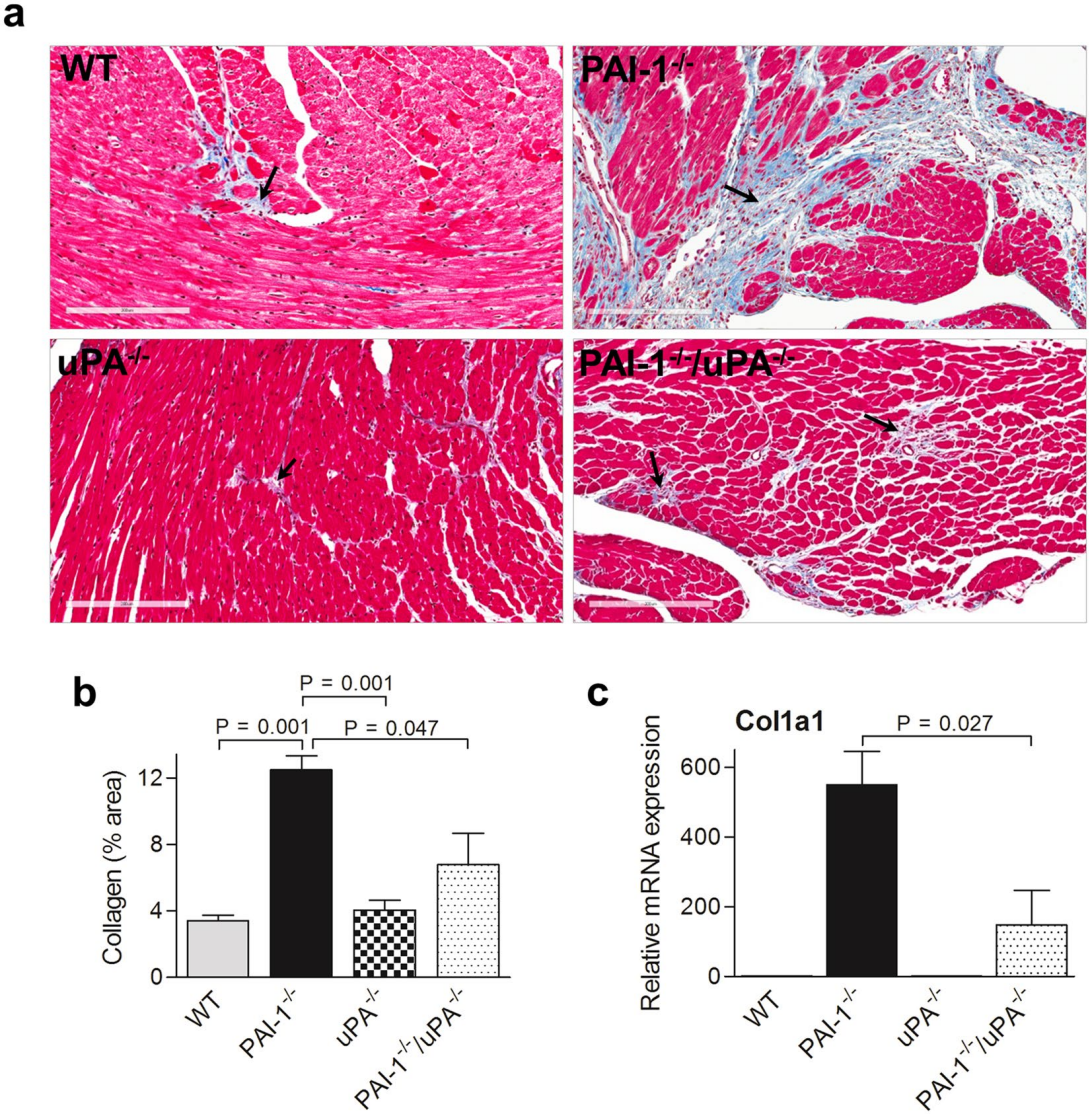
PAI-1^−/−^/uPA^−/−^ mice display reduced AngII-Ald-induced cardiac fibrosis. (**a**) Representative photomicrographs of Masson trichrome (collagen) stain (magnification: 200x). Extensive interstitial collagen deposition (blue staining) is indicated by black arrows. (**b**) Quantitative data showing the levels of collagen deposition in mouse hearts (n = 4–7 mice per group). (**c**) qRT-PCR analysis of cardiac Col1a1 gene expression (n = 4 mice per group).

Previous studies using PAI-1^−/−^ mice have shown that PAI-1 had a protective effect against cardiac fibrosis in both a spontaneous aging (nonhypertensive) model and a AngII infusion (hypertensive) model by maintaining microvascular integrity and cardiac architecture *in vivo* ^12–15^. This implied that uninhibited Pm generation may be a contributing factor in the progression of cardiac fibrosis. In the current study, a more severe hypertensive model (AngII-Ald infusion) was used to determine whether this cardioprotective effect of PAI-1 was still maintained in the absence of both uPA and PAI-1. To address this issue, PAI-1^−/−^/uPA^−/−^ mice were infused with AngII-Ald and the cardiac fibrosis response was compared to that observed in a single deficiency of PAI-1. WT and uPA^−/−^ mice were also employed for comparison. The blood pressure measurements in these mice showed a correlation between increased blood pressure with administration of AngII-Ald infusion (Supplementary Figure S1). Hearts from these mice were harvested at 4 wk of AngII-Ald challenge. The collagen content was then measured in the heart by both immunohistochemistry and qRT-PCR as an index of cardiac fibrosis. Masson trichrome staining for collagen showed increased deposition of collagen in the heart of PAI-1^−/−^ mice, and this staining was decreased in PAI-1^−/−^/uPA^−/−^ mice as compared to PAI-1^−/−^ mice (Fig. 1a,b). However, cardiac collagen content was minimal in WT and uPA^−/−^ mice. Similar observations were seen using picrosirius red staining (Supplementary Figure S2). The fibrosis severity in PAI-1^−/−^ mice was further confirmed by measuring the levels of plasma procollagen type III amino-terminal peptide (PIIINP), a marker of collagen turnover (Supplementary Figure S3). Similar results were observed with the mRNA level of collagen type I (Col1a1) (Fig. 1c).

The generation of myofibroblasts is associated with excessive collagen production, and is characterized as fibroblast-like cells that express α-smooth muscle actin (α-SMA). Histochemical analysis showing the number of α-SMA expressing cells from different groups is provided in Supplementary Figure S4. The data showed higher numbers of α-SMA expressing cells in the PAI-1^−/−^ cohort, indicating that the higher collagen deposition seen in PAI-1^−/−^ mice is due to more cells expressing collagen.

### AngII-Ald-induced vascular permeability and bleeding in PAI-1^−/−^/uPA^−/−^ mice

**Figure 2:**
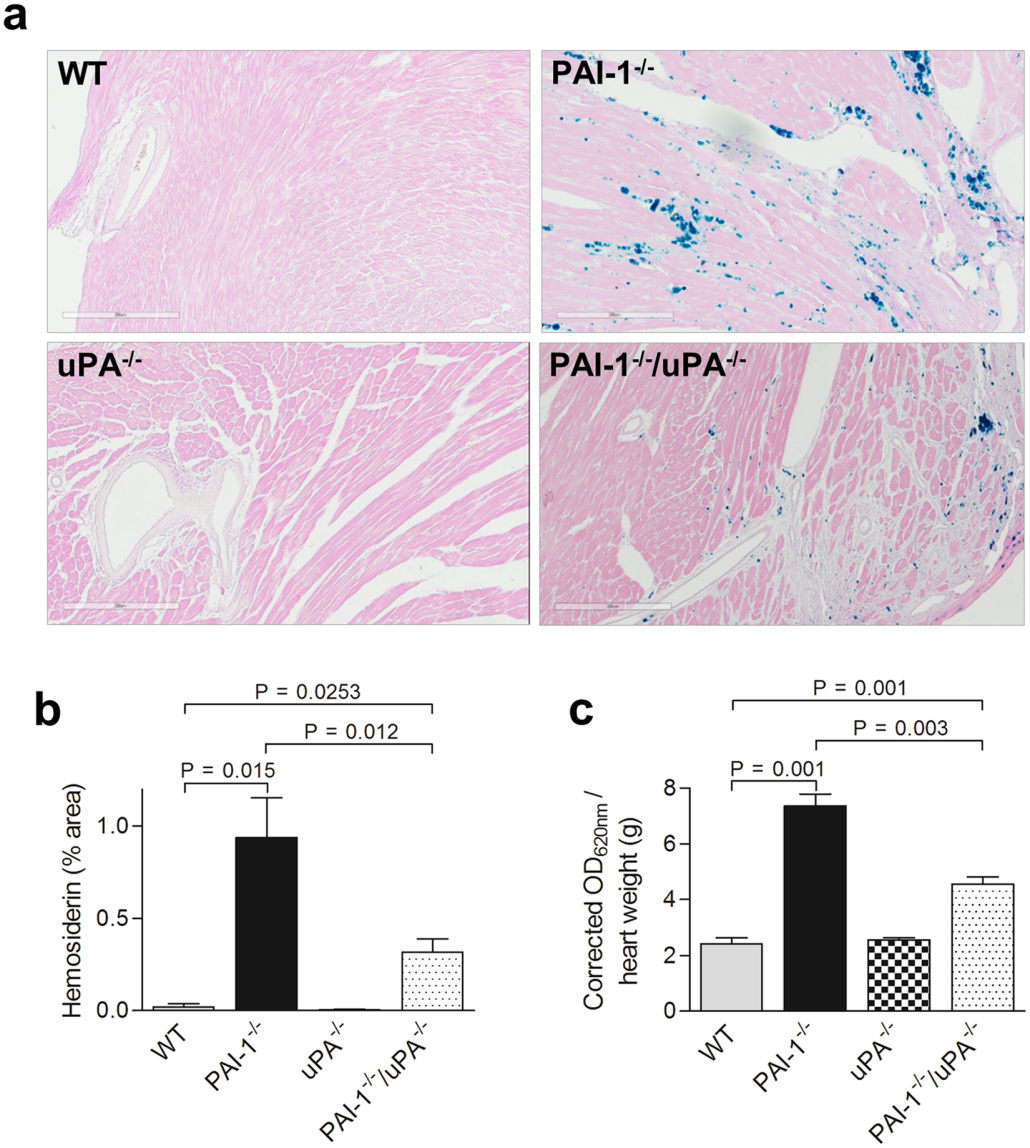
PAI-1^−/−^/uPA^−/−^ mice show decreased spontaneous bleeding and vascular permeability in AngII-Ald-infused mice. (**a**) Representative photomicrographs of heart sections from WT, PAI-1^−/−^, uPA^−/−^, and PAI-1^−/−^/uPA^−/−^ mice stained with Prussian blue for hemosiderin analysis at 4 wk of AngII-Ald infusion (magnification: 200x). (**b**) Histogram shows the quantification of areas of hemosiderin deposition in cardiac tissue (n = 4–7 mice per group). (**c**) Vascular permeability in mouse hearts at 1 wk of AngII-Ald infusion was detected by an Evans Blue vascular leakage assay (n = 3–6 mice per group).

It is hypothesized that early events of bleeding and inflammation are causative in the development of cardiac fibrosis^12^. In this study, hearts were stained with Prussian blue to examine interstitial hemosiderin granule deposition, indicative of prior bleeding. Abundant hemosiderin deposition was observed throughout the myocardium of AngII-Ald infused PAI-1^−/−^ mice (Fig. 2a,b). In contrast, hemosiderin deposition was significantly reduced in PAI-1^−/−^/uPA^−/−^ mice indicating the attenuation of bleeding in the myocardium. Hemosiderin deposition was nearly absent in AngII-Ald infused WT and uPA^−/−^ mice (Fig. 2a,b). To address whether the occurrence of interstitial haemorrhage in PAI-1^−/−^ mice correlates with cardiac vascular leakage, an *in vivo* Evans blue (EB) dye permeability assay was performed^12, 17^. As expected, PAI-1^−/−^ mice, after AngII-Ald infusion, displayed increased cardiac vascular leakage compared with PAI-1^−/−^/uPA^−/−^ mice (Fig. 2c).

### Local inflammation and leukocyte infiltration in PAI-1^−/−^/uPA^−/−^ hearts after AngII-Ald infusion

**Figure 3:**
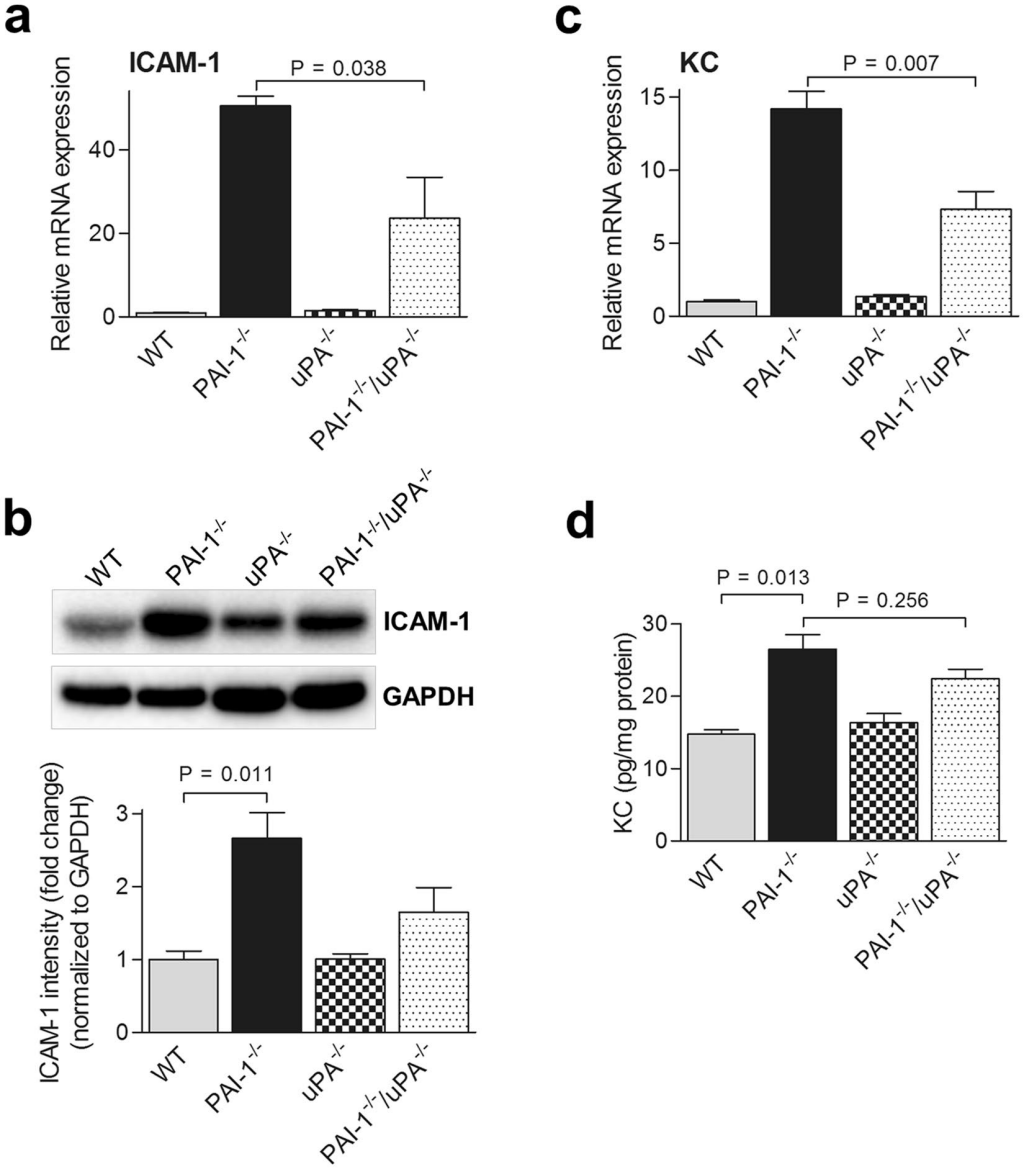
Inflammatory cytokine expression is attenuated in AngII-Ald-infused PAI-1^−/−^/uPA^−/−^ mice. qRT-PCR (**a**) and Western blot (**b**) analysis of ICAM-1 expression in heart tissues at 4 wk of AngII-Ald infusion. In Western blot, densitometric analysis of relative ICAM-1 expression after normalisation with GAPDH is shown in the lower panel. Western blot images are cropped for concise presentation. All uncut western blot images are provided in Supplementary Figure S6. qRT-PCR (**c**) and ELISA assays (**d**) of KC expression in heart tissues at 4 wk of AngII-Ald infusion were performed as described.

**Figure 4:**
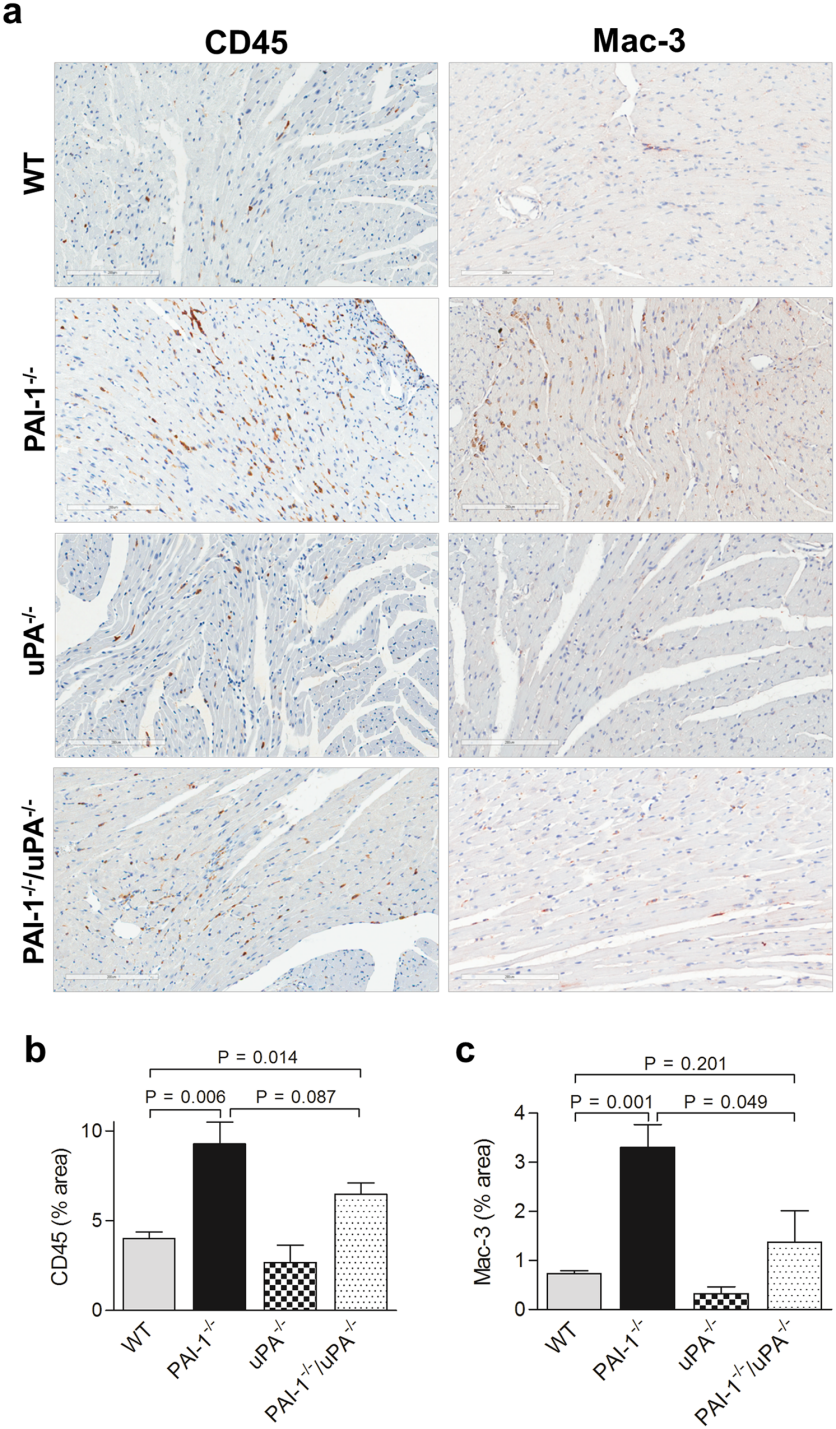
Leukocyte infiltration is altered in AngII-Ald-infused PAI-1^−/−^/uPA^−/−^ mice. (**a**) Representative photomicrographs from cardiac sections of AngII-Ald-infused (4 wk) mice immunostained with anti-CD45 and anti-Mac-3 antibody. CD45 positive leukocytes and Mac-3 positive macrophages are identified in brown. Magnification: 200x. (**b** and **c**) Quantification of CD45 and Mac-3 positive staining of hearts (n = 4–7 mice per group).

Microvascular leakage in hearts may provoke inflammation, and predispose to cardiac fibrosis^12^. To examine how a loss of uPA affects AngII-Ald induced inflammation in PAI-1^−/−^/uPA^−/−^ mice, we analysed cardiac mRNA levels encoding some key cytokines relevant to inflammation and cardiac fibrosis by qRT-PCR^12^. Intercellular adhesion molecule (ICAM-1), and keratinocyte-derived chemokine (KC) expression levels in cardiac tissues were significantly higher in PAI-1^−/−^ as compared to PAI-1^−/−^/uPA^−/−^ mice (Fig. 3). Infiltrating leukocytes were shown to contribute to the progression and pathogenesis of cardiac fibrosis^10, 12, 18^. To assess local leukocyte infiltration in cardiac tissue, CD45 and Mac-3/F480 staining and quantitation were performed. Leukocyte infiltration, as shown by positive CD45 and Mac3 staining, were extensively observed in PAI-1^−/−^ heart tissue (Fig. 4). In contrast, leukocyte and macrophage infiltration was significantly reduced in the tissue of treated PAI-1^−/−^/uPA^−/−^ mice while WT and uPA^−/−^ mice demonstrated little cardiac infiltration of leukocytes.

### Profibrotic gene expression in response to AngII-Ald infusion in PAI-1^−/−^/uPA^−/−^ mice hearts

**Figure 5:**
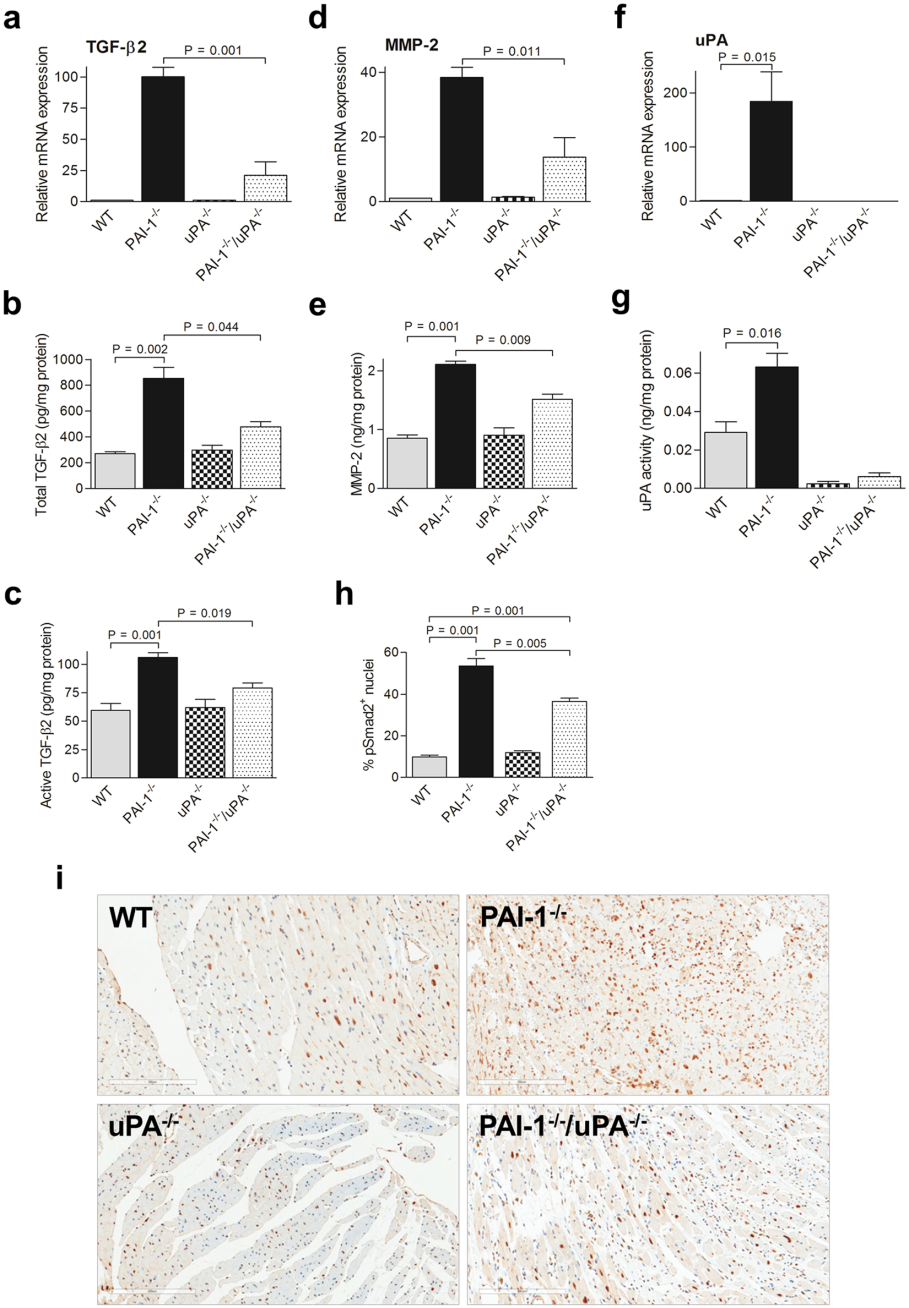
Cardiac ECM remodeling and Smad2 signaling are altered in AngII-Ald-infused PAI-1^−/−^/uPA^−/−^ mice. qRT-PCR analysis of mRNA transcripts of TGF-β2 (**a**), MMP-2 (**d**), and uPA (**f**), proteins associated with cardiac tissue remodeling and fibrosis, at 4 wk of AngII-Ald infusion (n = 4 mice per group). ELISA assays of total (**b**) and active TGF-β2 (**c**), MMP-2 (**e**), and active uPA (**g**) were performed as described. Immunohistochemistry (**i**) for pSmad2 in AngII-Ald-induced mice hearts and the quantitative data (**h**) showing the percent of cells with pSmad2^+^ nuclei (n = 3–4 mice per group).

TGF-β, a potent profibrotic molecule, has been shown to be elevated in cardiac tissue from PAI-1^−/−^ mice^12, 13^. In this study, it was demonstrated that both mRNA expression, and total and active TGF-β2 levels in PAI-1^−/−^/uPA^−/−^ mice were significantly lower in heart tissue than in that of PAI-1^−/−^ mice (Fig. 5a–c). Previous reports showed that matrix metalloproteinases (MMPs), and plasminogen activators (uPA and tPA) play an active role in fibrosis by regulating the release of factors associated with ECM that facilitate the synthesis of collagen, i.e., TGF-β^2, 12, 13, 19^. As shown in Fig. 5d, and Supplementary Figure S5, MMP-2, and tPA were upregulated after AngII-Ald infusion in PAI-1^−/−^ and to a lower extent, except for tPA, in PAI-1^−/−^/uPA^−/−^ mice. Consistently, higher uPA transcripts and activity were observed in the heart tissues of PAI-1^−/−^ as compared to WT mice (Fig. 5f,g).

### Smad2 signaling in PAI-1^−/−^/uPA^−/−^ mice

Overexpression of TGF-β is known to transduce signaling through its intracellular effectors, Smad2/3. Following translocation into the nucleus, Smad2/3 modulates the transcription of target genes that have been shown to be involved in cardiac fibrosis^13, 19–21^. To determine Smad2/3 signaling activation in cardiac tissue from AngII-Ald-infused mice, immunohistochemical analysis was performed using a phospho-specific Smad2 antibody. As expected, AngII-Ald infusion dramatically increased active phosphorylated Smad2 (p-Smad2) levels in PAI-1^−/−^ mice (Fig. 5h,i). However, the level of p-Smad2 was significantly diminished in PAI-1^−/−^/uPA^−/−^ mice. Minimal expression of p-Smad2 was observed in WT and uPA^−/−^ mice.

To determine if the myofibroblasts are the cells being activated by TGF-β and thus transduce Smad2/3 signaling, double staining of p-Smad2 and α-SMA by immunofluorescence was performed. Cells that express p-Smad2 but not α-SMA (data not shown) were observed. Consistent with this observation, the data shown in Figs 5h,i and S4 demonstrated more cells expressing p-Smad2 as compared to α-SMA in the PAI-1^−/−^ group, indicating that not all cells activated by TGF-β lead to myofibroblast formation.

### Effects of a deficiency of Pm activity on cardiac fibrosis in Pg^S743A/S743A^ and PAI-1^−/−^/Pg^S743A/S743A^ mice

**Figure 6:**
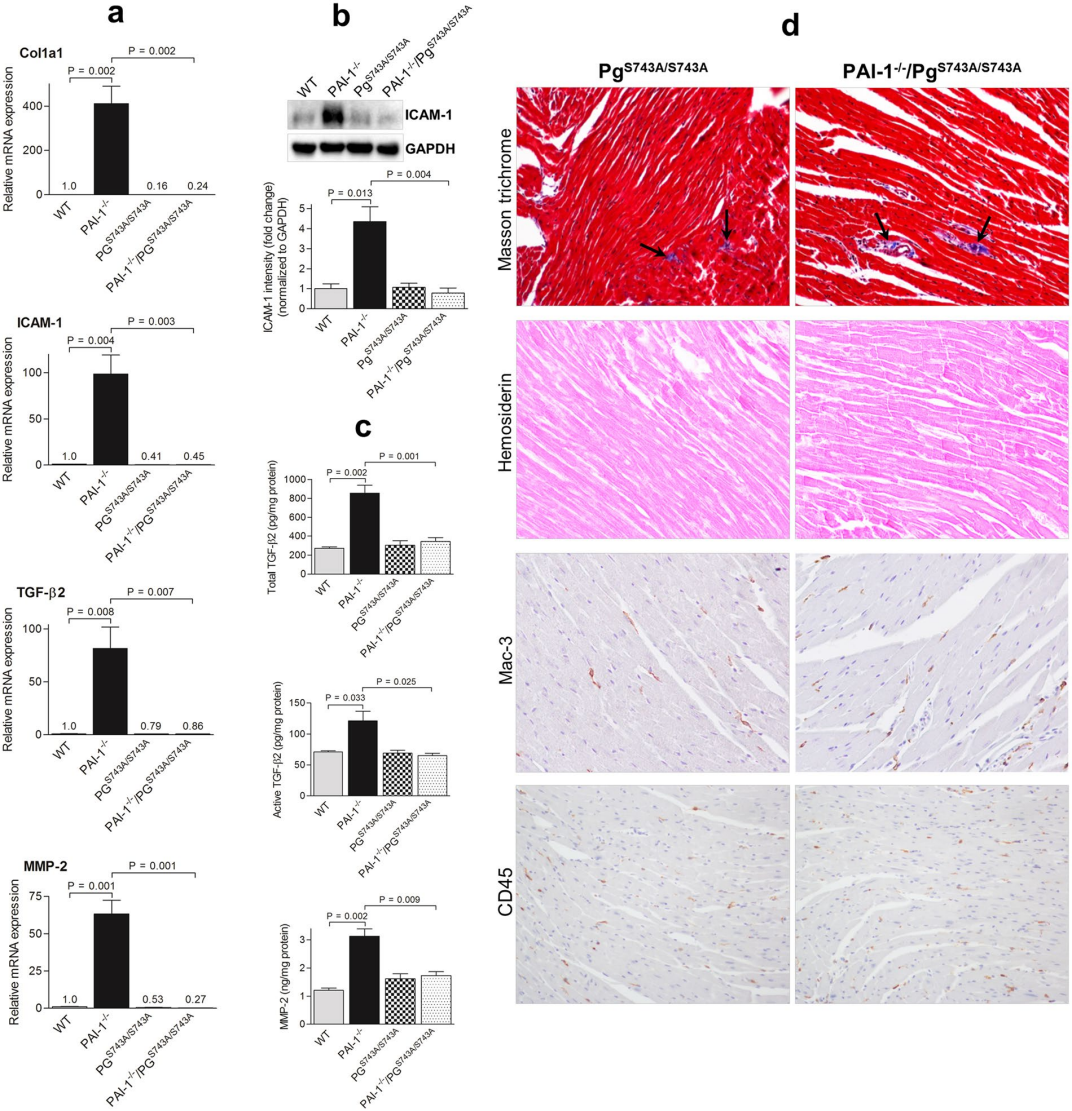
Loss of Pm activity results in reduced cardiac fibrosis in AngII-Ald infused mice. qRT-PCR (**a**) of transcripts of Col1a1, ICAM-1, TGF-β2, and MMP-2, Western blot analysis (**b**) of ICAM-1 expression, ELISA assays (**c**) of total and active TGF-β2 and MMP-2, and histological analyses (**d**) of Masson trichrome (collagen), hemosiderin deposition, and leukocyte infiltration (CD45 and Mac-3) at 4 wk of AngII-Ald infusion were performed as described (n = 3–4 mice per group). Western blot images are cropped for concise presentation. The uncut images are provided in Supplementary Figure S6.

Previous reports implicated Pm as playing a role in TGF-β activation^8, 22^. We hypothesized that elimination of Pm activity would inhibit TGF-β activation and thus suppress cardiac fibrosis, and that PAI-1^−/−^ mice that also lack the ability to express active Pm will reverse or severely ablate the cardiac phenotypes of PAI-1^−/−^ mice. We utilised Pg^S743A/S743A^ mice that express Pg at normal levels but generate an inactive Pm in order to define the role of active Pm in cardiac fibrosis^23^. The Pg^S743A/S743A^ mice were then cross-bred to generate the PAI-1^−/−^/Pg^S743A/S743A^ double-deficient mouse line. Both Pg^S743A/S743A^ and PAI-1^−/−^/Pg^S743A/S743A^ mice, after AngII-Ald infusion, had significantly lower mRNA and protein levels of Col1a1, ICAM-1, TGF-β2, and MMP-2 compared to PAI-1^−/−^ mice (Fig. 6a–c). Similarly, lower collagen deposition, bleeding, and leukocyte infiltration were seen in these mice as determined by histological analyses (Fig. 6d). Results from these studies implicate Pm as playing a major role in regulating cardiac fibrosis in this hypertensive model.

## Discussion

The role of the fibrinolytic system in a number of physiological and pathophysiological biologies has been extensively studied using both *in vitro* and *in vivo* models. However, its functional role in the development of cardiac fibrosis has remained elusive. In the present study, we utilised mice with single and double deficiencies of fibrinolytic proteins to decipher specific mechanisms that regulate hypertension-induced cardiac fibrosis in an AngII-Ald infusion mouse model. Our findings show that a total loss of PAI-1 leads to severe cardiac fibrosis. In addition, an absence of its direct target uPA or downstream product Pm can reduce the severity of cardiac fibrosis in PAI-1^−/−^ mice.

**Figure 7:**
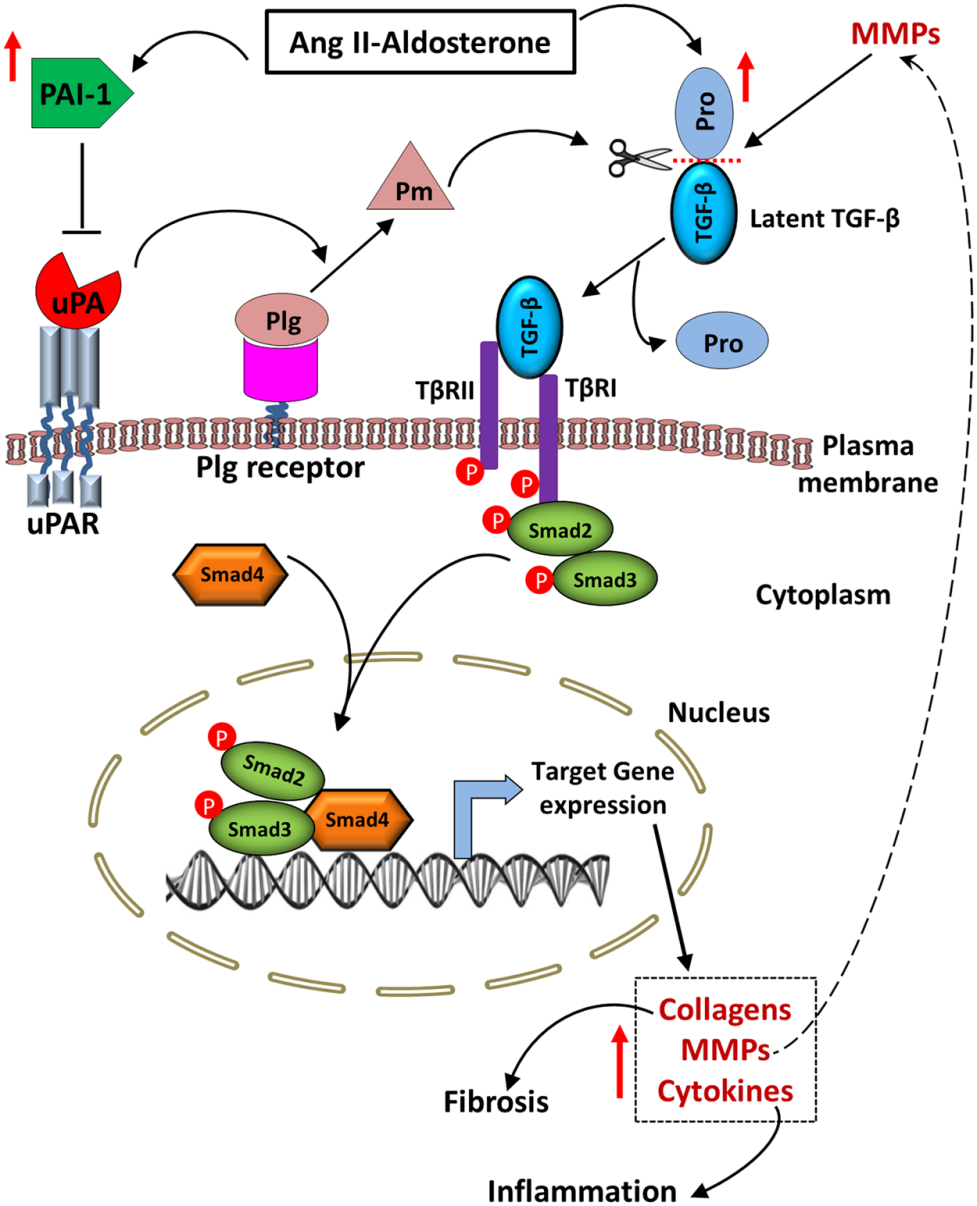
Schematic diagram showing functional roles of PAI-1 and uPA during AngII-Ald-induced cardiac fibrosis. AngII-Ald infusion in mice induces the expression of PAI-1 and TGF-β in the heart. Inhibition of TGF-β activation by PAI-1 inhibiting tPA/uPA generation of Pm suppresses TGF-β signaling in hearts. Binding of active TGF-β to its receptors (TGFβR) facilitates the recruitment of Smad2/3 to TGFβR leading to the phosphorylation of Smad2/3. Phosphorylated Smad2/3 forms a complex with Smad4 and translocates to the nucleus where it regulates transcription of target genes involved in cardiac tissue remodeling and fibrosis. Increased MMP activity in hearts further enhances the activation of TGF-β.

Factors that promote collagen synthesis and deposition in hearts play an important role in the development and progression of cardiac fibrosis. Pm is the key protease of the fibrinolytic system and can also proteolytically activate TGF-β. Activated TGF-β accelerates the Smad signaling thus driving the aberrant transcription of target genes (e.g. collagen) involved in cardiac tissue remodeling and fibrosis (Fig. 7). uPA and tPA are the primary physiological activators of Pg and therefore could regulate cardiac fibrosis. In contrast, PAI-1 plays a cardioprotective role by regulating Pm generation through inhibition of uPA and tPA. Two lines of evidence support the cardioprotective role of PAI-1 in response to AngII-Ald infusion in mice. First, PAI-1 expression increases significantly in AngII-Ald challenged hearts of WT and uPA^−/−^ mice relative to nonchallenged mice (data not shown)^24^ and these mice did not show any significant cardiac fibrosis. However, absence of PAI-1 in PAI-1^−/−^ and PAI-1^−/−^/uPA^−/−^ mice resulted in significant cardiac fibrosis. It is noted that the extent of cardiac fibrosis in PAI-1^−/−^/uPA^−/−^ was significantly diminished compared to PAI-1^−/−^ mice indicating that PAI-1 regulation of uPA activity confers cardiac protection against fibrosis. Since fibrosis was attenuated in this latter set of double deficient mice, but not eliminated, this implicated other targets of PAI-1, i.e., tPA, in regulating this disease. Consistent with these observations, PAI-1^−/−^ hearts showed increased expression of uPA (~180 fold) and tPA (~5 fold) as compared to WT mice after AngII-Ald infusion. Increased levels of uPA and tPA could induce excessive activation of TGF-β through enhanced Pm generation. Previous studies demonstrated that a tPA deficiency did not alter the development of cardiac fibrosis in AngII-induced hypertension in mice^14^. Consistent with this report, we observed no significant change in cardiac fibrosis in PAI-1^−/−^/tPA^−/−^ double deficient mice after AngII-Ald infusion (data not shown), indicating that unlike uPA, tPA plays a minimal role in the development of cardiac fibrosis.

It is assumed that the cardioprotective effect of PAI-1 is primarily mediated by regulation of Pm generation, and therefore a PAI-1^−/−^/Pg^S743A/S743A^ double-deficient mouse line expressing inactive Pm should reverse the cardiac fibrosis phenotype of PAI-1^−/−^. As expected, PAI-1^−/−^/Pg^S743A/S743A^ mice, after AngII-Ald infusion, showed complete reversal of cardiac fibrosis phenotypes observed in PAI-1^−/−^ mice, indicating that PAI-1’s cardioprotective effect is by regulating Pm generation.

Collectively, this study provides direct *in vivo* evidence that the mechanism underlying the cardioprotective effect of PAI-1 is through inhibiting uPA-mediated activation of Pg. These observations also provide new insights into the mechanisms associated with the development and progression of cardiac fibrosis and will serve to identify new therapeutic approaches toward regulating this disease.

## Methods

### General

All data are expressed as the mean ± SEM. The significance of experimental differences was evaluated by Student *t*-tests (GraphPad Software, Inc; La Jolla, CA). Values of *P* ≤ 0.05 were considered significant.

### Animal model

#### Ethics Statement

All animal studies were performed in accordance with the protocol titled “Induced Cardiac Fibrosis in Mice” (approval #14-03-1655). These protocols were reviewed and approved by the University of Notre Dame’s Institutional Animal Care and Use Committee (IACUC).

WT, PAI-1^−/−^, uPA^−/−^, and Pg^S743A/S743A^ mice, fully backcrossed to a C57BL/6 J background, have been described previously^23, 25, 26^. PAI-1^−/−^/uPA^−/−^ and PAI-1^−/−^/Pg^S743A/S743A^ double knockout mice were generated by crossing PAI-1^−/−^ with uPA^−/−^ and Pg^S743A/S743A^ single knockout mice, respectively. Eight to twelve wk old male mice were used for AngII-Ald infusion experiments. Under 1.5% isoflurane anaesthesia, an osmotic minipump (Alzet, Cupertino, CA) filled with AngII (Sigma, St. Louis, MO) and a pellet of D-aldosterone (0.1 mg for 21 days) (Innovative Research of America, Sarasota, FL) were implanted, subcutaneously, between the shoulder blades of mice to deliver AngII at an infusion rate of 2.75 μg/hr for 28 days. Consistent with previous studies^14, 27^, no pathological features were detected in the nontreated mice in all groups. Therefore, control groups of nonchallenged animals were not included in this study.

#### Quantitative real-time polymerase chain reaction (qRT-PCR)

Total RNA was extracted from the hearts using the RNeasy Mini kit (QIAGEN, Valencia, CA). The mRNA expressions were analysed by qRT-PCR using nonlabeled PCR primers and TaqMan® probes labeled at the 5′ end with 6-carboxyfluorescein (6-FAM) and BHQ-1 at the 3′ end (Supplementary Table 1) (Eurofins Scientific, Huntsville, AL). The reactions were carried out in 96-well plates using an ABI 7500 Fast Real-Time PCR system (Applied Biosystems, Foster City, CA). Amplification reactions were performed as follows: 30 min at 50 °C and 1 min at 95 °C, and 40 cycles of 15 sec at 95 °C and 1 min at 60 °C. Each sample was run in triplicate. The comparative C_T_ method (2^−∆∆CT^) was used to determine fold differences between the target gene and the reference gene (RPL-19).

#### Histological and immunohistochemical analysis

For histological analyses of cardiac tissue, mice were sacrificed by anaesthetizing with rodent cocktail (0.015 mg xylazine/0.075 mg ketamine/0.0025 mg acepromazine/g body weight). Hearts were fixed in paraformaldehyde/lysine/periodate (PLP), processed, and then paraffin embedded. Heart tissues were sectioned at 4 μm thickness for all histological studies. Masson’s trichrome, Picrosirius red, and Prussian blue staining were performed using standard procedures^12^. Immunohistochemical studies were performed as previously described^12^ using the following primary antibodies: monoclonal rat anti-mouse CD45 and Mac-3 (BD Biosciences, San Jose, CA), monoclonal mouse anti-αSMA (Sigma), and polyclonal rabbit anti-human pSmad2 (EMD Millipore, Billerica, MA). The secondary antibodies used were: HRP-conjugated donkey-anti-rabbit and donkey-anti-mouse IgG (Jackson ImmunoResearch, West Grove, PA), biotin-conjugated mouse-anti-rat IgG (BD Biosciences), and biotin-conjugated rabbit-anti-rat IgG (Dako). Biotin-conjugated secondary antibody-treated samples were further incubated with streptavidin-HRP (Biogenex, Fremont, CA). All sections were incubated with 3,3′-diaminobenzidine (DAB) (Vector Laboratories, Inc, Burlingame, CA) for colour development, followed by Hematoxylin QS nuclear counterstain (Vector Laboratories, Inc.). The slides were analysed with an Aperio Technologies CS Digital Slide Scanner (Leica Biosystems, Inc., Buffalo Grove, IL).

#### ELISA and western blotting

Tissue lysates were prepared from whole heart tissue in lysis buffer as described previously^28^. Protein concentration in the lysates was measured using a BCA Protein Assay Kit (Thermo Scientific, Rockford, IL). Concentrations of KC and MMP-2 proteins were determined by a murine ELISA kit (RayBiotech, Inc., Norcross, GA). uPA activity in heart tissue lysates was quantified using a mouse Urokinase ELISA kit (Molecular Innovations, Southfield, MI)^29^. Plasma PIIINP protein was determined by a murine ELISA kit (Cambridge, MA). Total TGF-β2 levels were measured using a mouse ELISA kit (antibodies-online, Atlanta, GA). The levels of active TGF-β2 were measured using a mouse TGF-β2 duo-set (DY7346) ELISA kit with an ancillary reagent pack according to the manufacturers’ instructions ((DY008, R&D Systems, Minneapolis, MN). The amount of active TGF-β2 was calculated using a standard curve generated from a known amount of recombinant mouse active TGF-β2 provided with the kit. Western blot analysis for ICAM-1 protein in tissue lysates as prepared above was performed as described^30^. Briefly, total protein (20 μg) was separated by SDS-gel electrophoresis, and then transferred to PVDF membrane. The membrane was immunoblotted with polyclonal goat-anti-mouse ICAM-1 (R&D Systems) overnight at 4 °C. The membrane was then washed and incubated with HRP-conjugated donkey-anti-goat IgG (Jackson ImmunoResearch). The resulting protein bands were visualised using the Clarity Western ECL kit (Bio-Rad, Hercules, CA) in a ChemiDoc MP system (Bio-Rad), and densitometric analysis was carried out using Image Lab software (Bio-Rad).

#### Evans blue (EB) dye assay

This assay was performed as previously described^12^. Anaesthesized mice were injected in the retro-orbital sinus with 2% EB dye (1 μl/g weight) one week after the pump and pellet were implanted. After 24 hr, mice were anaesthesized with rodent cocktail and sacrificed. The hearts were harvested, weighed, and incubated overnight in 0.6 ml formamide at 4 °C. The absorbance of the EB/formamide solution was measured at 620 nm.

## Electronic supplementary material

Supplemental Information
