# Supplementary material for: Plasminogen Activator Inhibitor-1 Protects Mice Against Cardiac Fibrosis by Inhibiting Urokinase-type Plasminogen Activator-mediated Plasminogen Activation
Source: Sci Rep. 2017 Mar 23;7:365. doi: 10.1038/s41598-017-00418-y (PMC5428408; doi:10.1038/s41598-017-00418-y)
Supplement: Supplementary file 1 — Supplemental Information [file 41598_2017_418_MOESM1_ESM.pdf]

Plasminogen Activator Inhibitor-1 Protects Mice Against Cardiac Fibrosis by Inhibiting Urokinase-type Plasminogen Activator-mediated Plasminogen Activation

Kamlesh K. Gupta, Ph.D.[1], Deborah L. Donahue, B.S.[1], Mayra J. Sandoval-Cooper, H.T.[1], Francis J. Castellino, Ph.D.[1,2], Victoria A. Ploplis, Ph.D.[1,2 *]

Supplemental Material

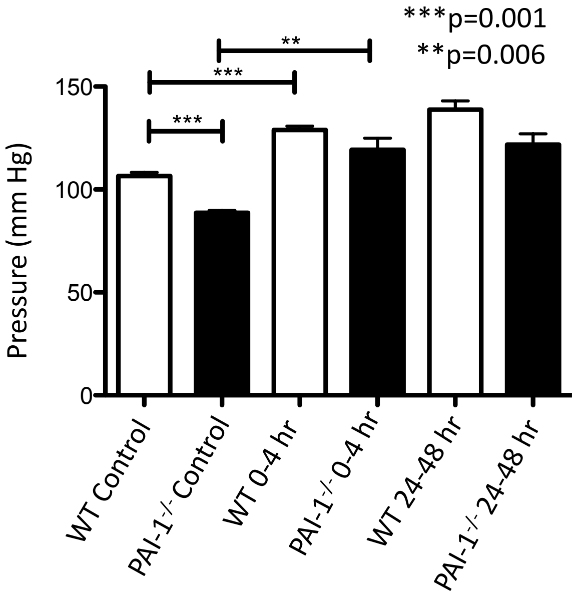

Figure S1. Blood pressure measurements in WT and PAI-$1^{-/-}$ mice.

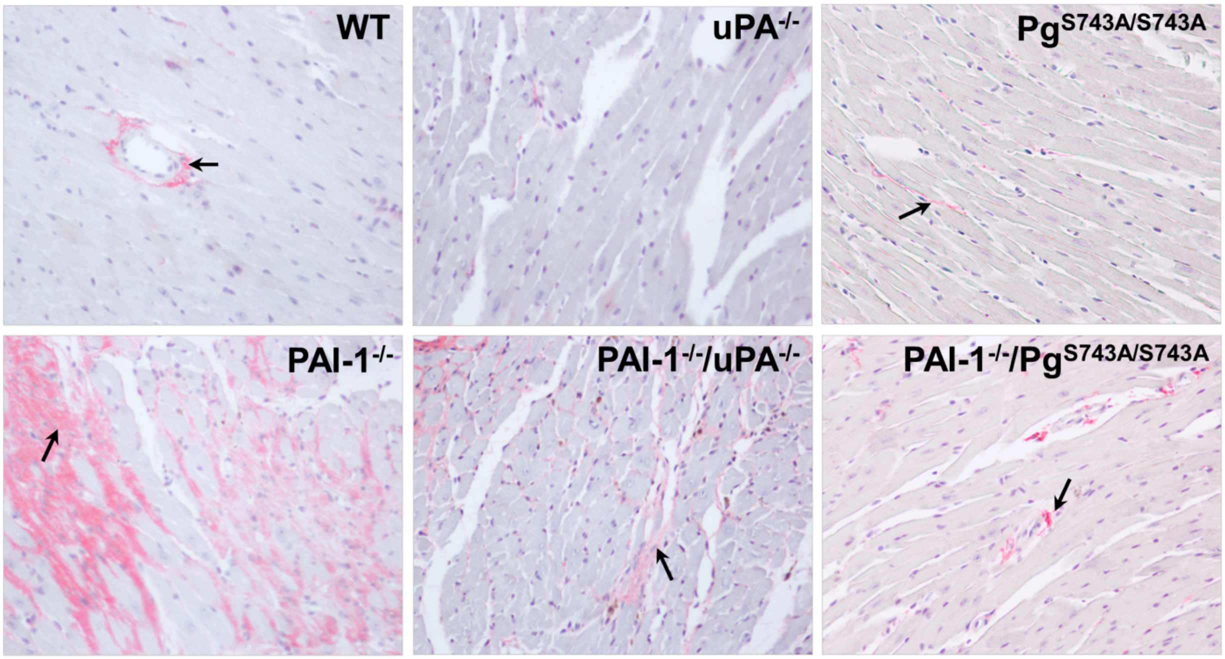

Figure S2. Histochemical analysis of collagen deposition in mouse hearts after 4 weeks of AngII-Ald infusion. Representative photomicrographs of picrosirius red (collagen) stain (magnification: 200x) are shown. Interstitial collagen deposition (pink/red staining) is indicated by black arrows.

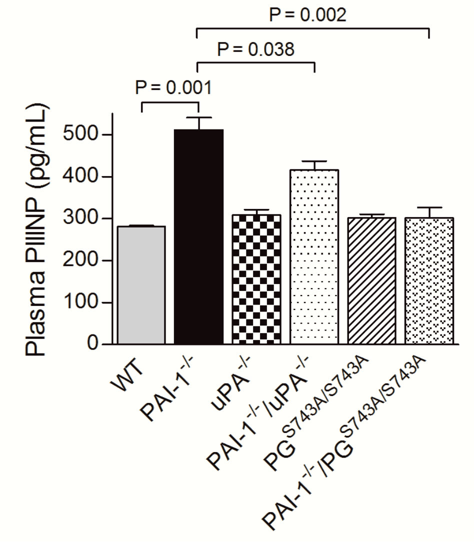

Figure S3. ELISA assays of plasma PIIINP. Experiment was performed as described. (n = 4 mice per group).

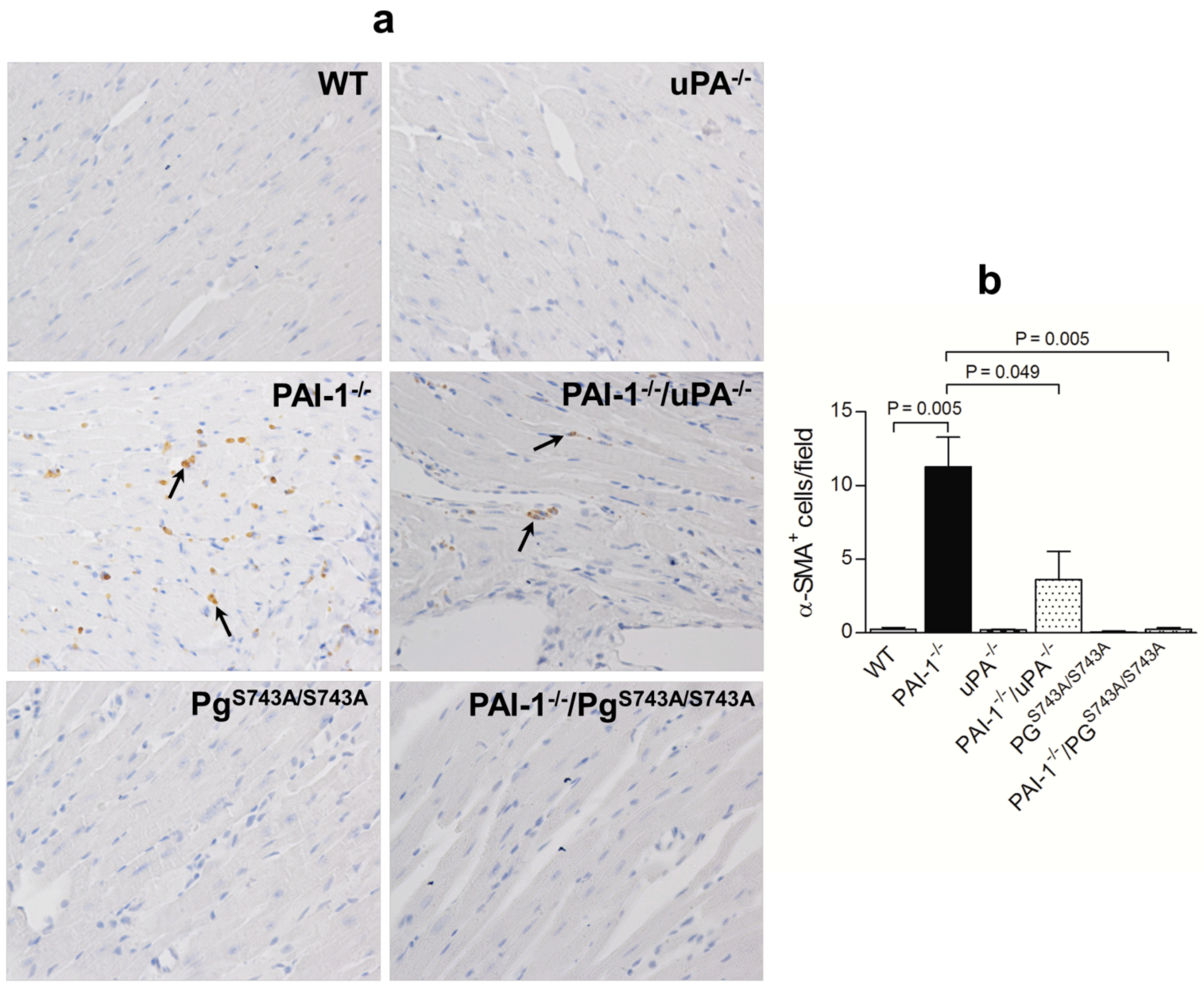

Figure S4. Histochemical analysis of α-SMA expression in mouse hearts after 4 weeks of AngII-Ald infusion. **(a)** α-SMA expressing cells (brown staining) are indicated by black arrows (magnification: 400x). **(b)** The panel represents the number of α-SMA expressing cells from different groups. The α-SMA-positive cells in eight fields (under 40x objective lens) from each heart sample were counted and plotted as the average number of cells per field. (n = 3 mice per group).

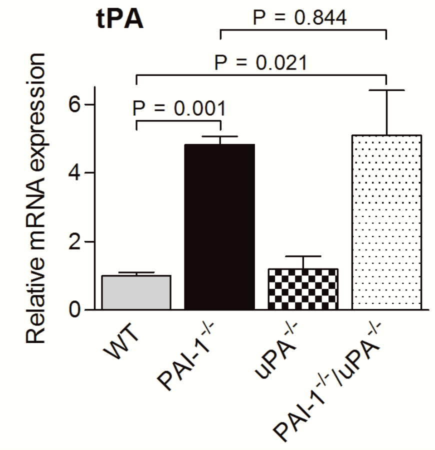

Figure S5. qRT-PCR analysis of cardiac tPA gene expression (n = 4 mice per group).

Figure 3b

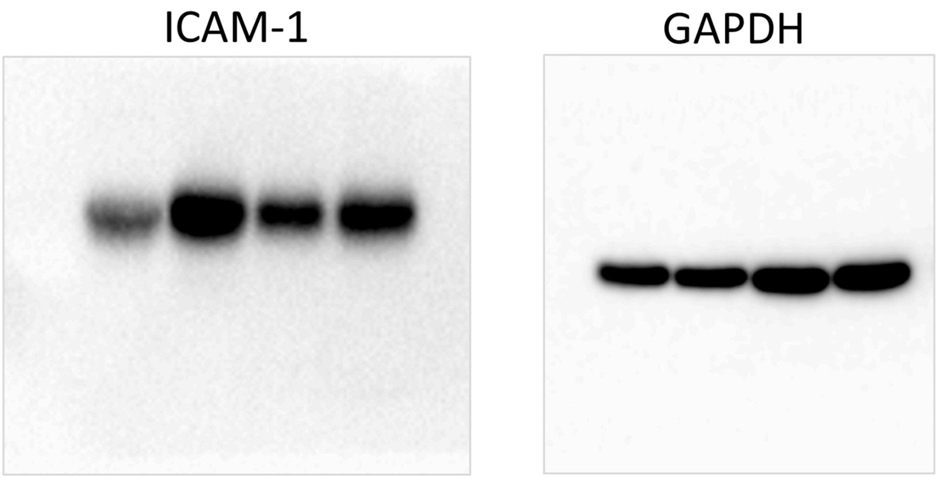

Figure 6b

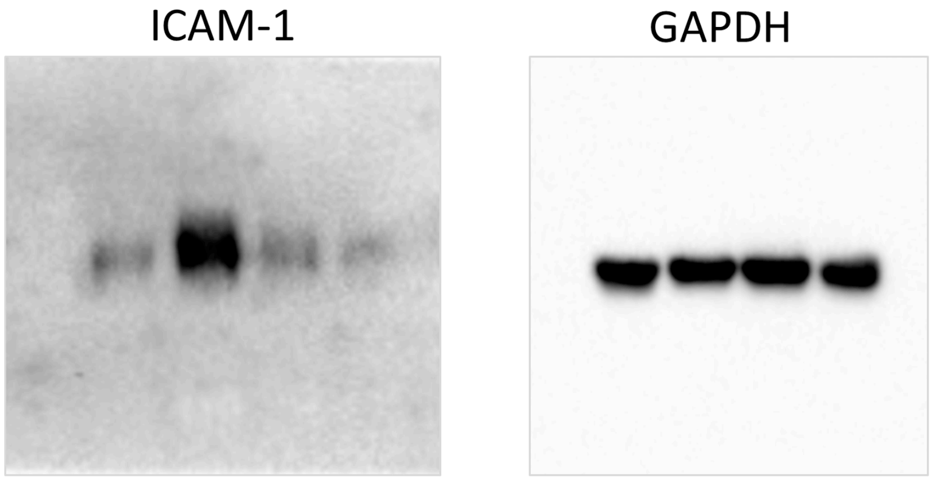

Figure S6. Uncut western blot images.

Table S1. List of murine gene specific primers and probes* for RT-PCR

Gene		Sequence (5' to 3')
RPL-19	Forward primer	ATGTATCACAGCCTGTACCTG
	Reverse primer	TTCTTGGTCTCCTCCTCCTTG
	Probe	TTTCGTGCTTCCTTGGTCTTAGACCT
PAI-1	Forward primer	TGCATCGCCTGCCATTG
	Reverse primer	GGACCTTGAGATAGGACAGTGCTT
	Probe	TGGAGGGTGCCATGGGCCA
uPA	Forward primer	TGCTATCATGGAAATGGTGACTCTT
	Reverse primer	GGGCATTGTAGGGTTTCTGAAG
	Probe	ACACTGATACCAAAGGTCGGCCCTGC
tPA	Forward primer	GCCAACCTTCCATAACTCTAGGATT
	Reverse primer	GTTGAGACATGATCTTGTGGTTCAGT
	Probe	CCAAAGTCTGCCCTCCTGGTCCAC
TGF-β2	Forward primer	ACCTTTTTGCTCCTGCATCTG
	Reverse primer	GTCGAGGGTGCTGCAGGTA
	Probe	TCCCGGTGGCGCTCAGTCTGT
Col1a1	Forward primer	TCCCTGGAATGAAGGGACAC
	Reverse primer	GCTCTCCCTTAGGACCAGCA
	Probe	CCAGCATCTCCTTTGGCACCA
MMP-2	Forward primer	TTCCAGGGCACCTCCTACAA
	Reverse primer	CCATATTTCTTATCCCGGTCATAGTC
	Probe	CCGCACCGACGGCTACCGC
ICAM-1	Forward primer	GGAGGTGGCGGGAAAGTT
	Reverse primer	AGGTCCAGTTCCCCAAGCA
	Probe	CGTGCTGTATGGTCCTCGGCTGGA
KC	Forward primer	CTGGGATTCACCTCAAGAACATC
	Reverse primer	CAGGGTCAAGGCAAGCCTC
	Probe	TTGCCCTCAGGGCCCCACTG

*Probes were labeled at the 5'-end with the reporter dye, 6FAM, and at the 3'-end with the quencher dye, BHQ1a (Eurofins Scientific, Huntsville, AL).
